# Tacrolimus Improves Therapeutic Efficacy of Umbilical Cord Blood-Derived Mesenchymal Stem Cells in Diabetic Retinopathy by Suppressing DRP1-Mediated Mitochondrial Fission

**DOI:** 10.3390/antiox12091727

**Published:** 2023-09-06

**Authors:** Hang Hyo Jo, Yeong Seok Goh, Hye Jih Kim, Dae Hyun Kim, Hyemin Kim, Jiyi Hwang, Ji Seung Jung, Nanyoung Kang, Sang Eun Park, Kyung Mee Park, Hyun Jik Lee

**Affiliations:** 1 Laboratory of Veterinary Physiology, College of Veterinary Medicine and Veterinary Medicine Center, Chungbuk National University, Cheongju 28644, Republic of Korea; hyo1126@chungbuk.ac.kr (H.H.J.);; 2 Institute for Stem Cell & Regenerative Medicine (ISCRM), Chungbuk National University, Cheongju 28644, Republic of Korea; 3 Laboratory of Veterinary Surgery and Ophthalmology, College of Veterinary Medicine, Chungbuk National University, Cheongju 28644, Republic of Korea

**Keywords:** diabetic retinopathy, mesenchymal stem cells, tacrolimus, mitochondrial dynamics, O-GlcNAc transferase, apoptosis

## Abstract

Diabetic retinopathy (DR) is a leading cause of blindness in diabetic patients. Umbilical cord blood-derived mesenchymal stem cells (UCB-MSCs) are emerging as a promising new drug for degenerative disease associated with diabetes. Recent studies have shown that high glucose-increased excessive calcium levels are a major risk factor for mitochondrial reactive oxygen species (mtROS) accumulation and apoptosis. This study aimed to investigate the role of high glucose-induced NFATC1 signaling in mitochondrial oxidative stress-stimulated apoptosis and the effect of tacrolimus on the therapeutic efficacy of subconjunctival transplantation of UCB-MSCs in a DR rat model. High glucose increased mtROS and cleaved caspase-9 expression in UCB-MSCs. High glucose conditions increased O-GlcNAcylated protein expression and nuclear translocation of NFATC1. Tacrolimus pretreatment recovered high glucose-induced mtROS levels and apoptosis. In the DR rat model, subconjunctival transplantation of tacrolimus-pretreated MSCs improved retinal vessel formation, retinal function, and uveitis. In high glucose conditions, tacrolimus pretreatment reduced protein and mRNA expression levels of DRP1 and inhibited mitochondrial fission. In conclusion, we demonstrated that high glucose-induced O-GlcNAcylation activates NFATC1 signaling, which is important for DRP1-mediated mitochondrial fission and mitochondrial apoptosis. Finally, we proposed NFATC1 suppression by tacrolimus as a promising therapeutic strategy to improve the therapeutic efficacy of UCB-MSC transplantation for DR treatment.

## 1. Introduction

Diabetic retinopathy (DR) is a major cause of blindness in patients with diabetes that interferes with the normal interaction of the retinal nerves and vascular components, resulting in vascular permeability, neovascularization, and loss of nerve function [1]. Although vascular endothelial growth factor (VEGF) inhibition or laser therapy has been developed to treat DR, it is limited in that it slows the progression of the disease but cannot restore visual function [2]. Therefore, new treatments are being developed to prevent or slow the development and progression of DR. Mesenchymal stem cells (MSCs) are multipotent stromal cells that originate from many connective tissues and can differentiate into different cell types, and MSCs are important for the preservation of tissue homeostasis and have regenerative potential. Therefore, it is a promising tool for tissue regeneration [3,4]. However, because MSCs are easily affected by the surrounding microenvironment, the effect of MSCs is unstable, and for defective tissues, MSCs are greatly affected by the surrounding oxidative stress environment [5]. High glucose conditions increase the expression of aging markers and inhibit MSC proliferation [6]. In addition, the expression of the mitochondrial functional regulatory genes *COX1*, *PGC-1a*, *ND2*, *ND9*, and *TFAM* in MSCs under high glucose conditions decreases, which causes excessive oxidative stress [7]. Consistently high glucose concentrations change the latent regenerative power of MSCs, thereby decreasing the survival rate of MSCs [8]. Therefore, further studies are required to elucidate the mechanisms that impair MSCs function in hyperglycemia.

Diabetes is closely associated with calcium (Ca^2+^), which is an essential signaling molecule that regulates a variety of biological functions. A previous study showed that insulin is secreted in a Ca^2+^-dependent manner and that diabetic patients contribute higher serum Ca^2+^ than non-diabetic patients [9]. In addition, several studies have shown that excess Ca^2+^ impairs mitochondrial function and membrane potential in SH-SY5Y cells, a human neuroblastoma cell line, and plays an important role in mitochondrial-dependent apoptosis [10]. A continuous increase in intracellular Ca^2+^ levels breaks down the mitochondrial membrane potential (MMP) and causes cellular energy supply problems, which results in mitochondrial dysfunction [11]. Mitochondrial dysfunction due to cellular stress, such as oxidative stress, is a major cause of accelerated aging, apoptosis induction, and reduced rate in MSCs [12]. However, no specific studies have reported the changes in Ca^2+^ levels in MSCs under high glucose conditions and the effects of intracellular Ca^2+^ regulation on MSC transplantation into a DR model. Regarding the excessive Ca^2+^ due to hyperglycemia that causes mitochondrial dysfunction of MSCs, correction of the impaired Ca^2+^ homeostasis may improve the therapeutic efficiency of MSC-based treatment for DR. 

Tacrolimus (FK506) is an immunosuppressant that binds to immunophilin FKBP12. The FK506-FKBP12 complex binds with calcineurin to inhibit phosphatase activity, thereby inhibiting the nuclear transposition of the nuclear factor of activated T cells (NFAT) [13]. It has been reported that the aberrant activation of intracellular Ca^2+^ signaling by STIM-1 and Orai-1-mediated Ca^2+^ influx plays a key role in the Ca^2+^-calcineurin-NFAT axis activation, which is closely associated with the etiology of autoimmune diseases [14]. In addition, a previous study reported that tacrolimus lowered TNF-a, VEGF, iNOS, and COX-2 in streptozotocin-induced diabetic mice and prevented early retinal neovascularization in mouse eye tissues by weakening the diabetes-related neovascularization, activation of NF-κB, and retinal inflammation [15]. Due to these characteristics, neural regeneration studies using tacrolimus and MSCs with their excellent regenerative effects were conducted, and neurite extension was enhanced when nerve transplantation was performed with MSCs in combination with tacrolimus treatment [16]. However, the effect and mechanism of action of tacrolimus in the transplantation of MSCs in a DR model remains poorly understood. As previous studies with tacrolimus and MSCs have used ocular or systemic administration of tacrolimus to suppress cell transplant rejection or to enhance MSC therapy, the potential risk of systemic immunosuppressive side effects with tacrolimus remains [15,17].

Many preclinical studies have been published on the therapeutic effect of stem cells in DR. In some studies with intravitreal injection, the protective effects, such as prevention of retinal capillary dropout, loss of ganglion cells, oxidative damage, neovascularization, and visual acuity improvement, were demonstrated [17,18,19,20,21]. On the other hand, some studies reported negative results, such as an increase in pericyte loss, retinal vessel atrophy, cataract formation, and retinal inflammatory response [22,23]. Because the therapeutic effect of intravitreal stem cell injection remains controversial, umbilical cord-derived MSCs (UCB-MSCs) were subconjunctivally injected to confirm their safety and therapeutic effect on DR in this study. Therefore, we investigated the role of high glucose-induced NFATC1 signaling in mitochondrial apoptosis and confirmed the effect of tacrolimus on the therapeutic efficacy of subconjunctival transplantation of UCB-MSCs in a DR rat model.

## 2. Materials

UCB-MSCs, fetal bovine serum (FBS), and high glucose CEFOgr human MSC growth medium culture medium were purchased from CEFO (Seoul, Republic of Korea). Phosphate-buffered saline (PBS) and MEM alpha (α-MEM) medium modification were obtained from Hyclone (Logan, UT, USA). O-GlcNAc, Bax, Bcl-2, DRP1, FIS1, and β-actin antibodies were acquired from Santa Cruz Biotechnology (Dallas, TX, USA). O-GlcNAc transferase (OGT), O-GlcNAcase (OGA), p-NFATC1, NFATC1/NFAT2, β-tubulin, and lamine A/C antibodies were purchased from Novus Biologicals (Littleton, CO, USA). p-DRP1(Ser616) and cleaved caspase-9 antibodies were purchased from Cell Signaling Technology (Beverly, MA, USA). D-glucose, L-glucose, N-Acetyl-D-glucosamine, antimycin A, carbonyl cyanide 3-chlorophenylhydrazone (CCCP), ionomycin, BAPTA-AM, and MitoTEMPO were obtained from Sigma-Aldrich (St. Louis, MO, USA). Mdivi-1 and ethylene-glycoltetraacetic-acid (EGTA) were purchased from Cayman Chemical (Ann Arbor, MI, USA).

### 2.1. Cell Culture 

UCB-MSCs were cultured in high glucose CEFOgro Human MSC Growth Medium (CEFO, Seoul, Republic of Korea), including 10% FBS and 0.5% antibiotics (penicillin and streptomycin). The cells were incubated at 37 °C with 5% CO_2_. When the cells had grown to approximately 60–70% confluency, the growth medium was changed to serum-free α-MEM medium (Hyclone, Logan, UT, USA) for 24 h.

### 2.2. Trypan Blue Exclusion Assay 

UCB-MSCs were washed with PBS and incubated in a 0.05% trypsin solution. Trypsin inhibitor was added to the cells, and they were suspended. Suspended cells were placed on ice and stained with 0.4% trypan blue (Sigma-Aldrich). To determine cell viability, the cells were counted using cell counting chamber slides (Thermo Fisher, Waltham, MA, USA). Trypan blue-stained cells were dead, and the unstained cells were live. Cell viability = {1 − (number of trypan blue-stained cells/number of total cells) × 100}.

### 2.3. Measurements of Mitochondrial Reactive Oxygen Species and Membrane Potential

To detect the generation of mitochondrial reactive oxygen species (mtROS) and membrane potential, MitoSOX mitochondrial superoxide indicator (Thermo Fisher) and TMRE (Sigma-Aldrich) were used. Cells were washed with PBS and incubated with 10 μM MitoSOX or 200 nM tetramethylrhodamine, ethyl ester (TMRE) in fluorobrite (Thermo Fisher, Waltham, MA, USA) at 37 °C for 15 min. The cells were washed again with PBS twice. Fluorescence intensities of mitochondrial ROS or mitochondrial membrane potential were detected using a microplate reader at excitation and emission wavelengths of 485 and 535 nm, respectively.

### 2.4. Lactate Dehydrogenase Cytotoxicity Assay

To detect the generation of lactate dehydrogenase (LDH), an LDH release assay kit (EZ-LDH, DoGenBio, Seoul, Republic of Korea, DG-LDH500) was used. UCB-MSCs were then seeded at a density of 1.5 × 10^4^ cells/well in a 96-well plate. The plate was centrifuged at 600× *g* for 5 min, and 50 μL of the cell supernatant was mixed with the LDH reaction mixture (100 μL). The optical density was measured using a microplate reader at 450 nm.

### 2.5. Western Blot Analysis

Samples were lysed with RIPA buffer (Atto, Tokyo, Japan), and the cells were crushed with a sonicator to extract the proteins. To remove cellular debris, the extracted protein samples were centrifuged for 10 min at 12,000× *g,* and for the protein quantification, a BCA assay (Thermo Scientific) was used. Proteins were resolved by SDS-PAGE and transferred to polyvinylidene fluoride (PVDF) membranes. The transferred PVDF membranes were blocked with 5% skim milk (LPS solution, Daejeon, Republic of Korea) and probed with the primary antibody overnight at 4 °C. After washing three times with tris-buffered saline containing 0.1% Tween-20 (TBST) solution, the blots were incubated with the secondary antibodies for 2 h at room temperature. The PVDF membranes were washed three times with TBST, and the secondary antibodies were conjugated with horseradish peroxidase (Bio-Rad, Hercules, CA, USA). Full-length gel images are presented in the Supplemental Information (Supplementary file: Figures S2–S4).

### 2.6. Subcellular Fractionation

To perform nuclear fractionation, the Ez Subcell kit (Atto, Tokyo, Japan) was used. Cells were vortexed with the fraction buffer mixture and incubated for 10 min at 4 °C. The cells were broken by shear pipetting to form the lysate. The lysates were centrifuged at 700× *g* for 10 min at 4 °C. The supernatant, as the cytosol fraction, was removed and further centrifuged at 12,000× *g* for 10 min at 4 °C to obtain the clear cytosol supernatant, and the pellet, as a nuclear fraction, was crushed using a sonicator to extract the proteins.

### 2.7. Immunocytochemistry

Cells were cultured in confocal dishes and fixed with acetone (Sigma-Aldrich) for 10 min. The fixed cells were washed three times with PBS containing 0.1% Tween-20 (PBST). The cells were blocked with 5% bovine serum albumin (BSA) (LPS solution, Daejeon, Republic of Korea) in PBST for 30 min. The cells were incubated with the primary antibodies in PBST for 1 h at room temperature. The cells were then washed with PBST three times for 5 min each time, and the cells were incubated with the secondary antibody, Alexa Fluor 488 (Thermo Fisher), for 30 min. After washing with PBST three times, the cells were incubated with 4′,6-Diamidino-2-Phenylindole, dihydrochloride (DAPI) (Thermo Fisher). Immune-stained cells were visualized by a super-resolution radial fluctuation imaging system (Andor Technology, Belfast, UK).

### 2.8. Mitochondrial Morphology Analysis

The cells were cultured in confocal dishes and incubated with Mitotracker Green FM (200 nM) for 30 min at 37 °C incubator. After incubation, the cells were washed with PBS three times. The quantitative analysis of mitochondrial morphology was conducted using the Image J software (developed by Wayne Rasband, National Institutes of Health, Bethesda, MD, USA; https://imagej.nih.gov/ij/download.html (accessed on 5 November 2022)). The shape of individual mitochondrial particles was analyzed using the methods described in the previous study [24].

### 2.9. RNA Isolation and Reverse Transcription Polymerase Chain Reaction

To obtain the RNA samples, an RNA extraction kit (TaKaRa Biomedical, Otsu, Japan) was used. A 1 μg/μL concentration of the extracted RNA was prepared. To produce cDNA, reverse transcription polymerase chain reaction (RT-PCR) was performed using a Maxime RT-PCR premix kit (iNtRON Biotechnology, Sungnam, Republic of Korea). Reverse transcription was performed for 1 h at 45 °C followed by 5 min at 95 °C.

### 2.10. Real-Time Quantitative PCR

The cDNA sample was amplified with a TB Green Premix Ex Taq (TaKaRa Biomedical, Otsu, Japan) kit. The mRNA expression levels of the *DRP1*, *FIS1*, *MFN1*, *MFN2*, *OPA1*, and *ACTB* genes were determined using the CFX connect real-time PCR detection system (Bio-Rad, Hercules, CA, USA) with 1 μg of cDNA sample and mRNA primers mixture. The gene expression level was normalized to the mRNA expression of *ACTB*. Real-time PCR was performed for 10 min at 95 °C for DNA polymerase activation and 40 cycles of 15 s at 94 °C, 20 s at 55 °C, and 30 s at 72 °C. The primer sequences are described in Supplemental Information (Supplementary file: Table S1).

### 2.11. Measurement of Ca^2+^ Influx

The levels of cellular Ca^2+^ level were measured using Fluo-3, AM (Thermo Fisher). The cells were washed with PBS and incubated with 2 μM Fluo-3, AM solution in a serum-free medium at 37 °C for 20 min. The cells were washed again with PBS twice. The Fluo-3, AM-positive cells were detected using a plate reader at an excitation and emission wavelength of 485 and 528 nm, respectively.

### 2.12. Animals

All animal studies were reviewed and approved by the Institutional Animal Care and Use Committee (CBNUA-1613-21-01) at the Chungbuk National University and in accordance with the Association for Research in Vision and Ophthalmology Statement for the Use of Animals in Ophthalmic and Vision Research. Thirty-eight male Spraque–Dawley rats were obtained from Samtako Bio Korea Laboratories (Osan, Republic of Korea) at 8 weeks of age. The animals were maintained in a conventional environment with a standard 12 h day/12 h night cycle in experimental animal facilities at Chungbuk National University and fed a normal pellet chow diet (Experimental Rat & Mouse diet, Purina, St. Louis, MO, USA). After 7 days of acclimatization, diabetes was induced by the intraperitoneal injection of a freshly prepared solution of streptozotocin (STZ) in citrate buffer (pH 4.5) at 55 mg/kg after fasting for 8 h [25,26,27,28]. The same amount of citrate buffer was intraperitoneally injected into the control group (nine rats, 18 eyes). Diabetes was confirmed ten days after STZ injection with blood glucose levels higher than 250 mg/dL using a commercial kit (Fora G11, ForaCare, Moorpark, CA, USA). Five rats were used as intact control. Twenty-nine diabetic rats were double-blind and randomly divided into three groups: the DR group (DR, ten rats, 20 eyes), MSC injection group (MSC, nine rats, 18 eyes), and tacrolimus-pretreated MSC injection group (T-MSC, ten rats, 20 eyes). Four rats were excluded due to failure to develop diabetes. The order of injection was randomly performed. We decided on the sample size according to the previous references [29].

### 2.13. Preparation and Injection of MSCs and T-MSCs

Tacrolimus (100 nM in dimethyl sulfoxide; DMSO, Sigma, St. Louis, MO, USA) was pretreated in culture media for 24 h, followed by washing and then for a T-MSC group injection. The same amount of DMSO was added in culture media for the same period for an MSC group injection. Subconjunctival MSCs (1 × 10^5^ MSCs in 30 µL phosphate-buffered saline; PBS) and pretreated MSCs injections were performed with a 31G insulin syringe (Ultra-Fine II short needle, BD biosciences, Franklin Lakes, NJ, USA) under topical anesthesia (Alcaine, Alcon, Geneva, Switzerland), every two weeks from two weeks after the STZ injection. In control and DR groups, 30 µL of PBS was injected. A total of 4 injections were performed for 8 weeks. All injection procedures were performed in experimental animal facilities at Chungbuk National University.

### 2.14. Ophthalmic Examination

Nine weeks after the STZ injection, all rats in the study underwent ophthalmic examinations. A slit lamp exam (MW50D, Shigiya Machinery Works, Hiroshima, Japan) was used to evaluate the anterior segment. Indirect microscopy (28D double aspheric lens, Volk Optical, Mentor, OH, USA) was used for the fundus evaluation and flash and flicker (8.0 cd·s/m^2^, 2 Hz and 28.3 Hz, respectively) electroretinography (ERG, RETevet, LKC, Gaithersburg, MD, USA) was used for the retinal function evaluation ad they were performed after dilating pupil with topical 0.05% tropicamide and phenylephrine (Mydrin-P, Santen, Osaka, Japan).

### 2.15. Histologic Examination

After enucleation, the eyes were preserved overnight in Modified Hartmann/Davidson Formalin (BioFix HD, BioGnost, Zagreb, Croatia). The eyes were divided in two along the median plane, the lenses were removed, and then standard tissue processing was performed. Serial 5μm paraffin retinal sections were cut and stained using hematoxylin and eosin. Retinal cell layers were grouped into 4 groups: (1) nerve fiber layer and ganglion cell layer (NFL + GCL); (2) inner nuclear layer and inner plexiform layer (INL + IPL); (3) outer nuclear layer and outer plexiform layer (ONL + OPL); and (4) photoreceptor layer (PRL), except for the retinal pigment epithelium layer. The thickness of each group and the entire retina were measured, and the ratios were evaluated. Immunohistochemistry staining was performed using a mouse and rabbit Specific HRP/DAB (ABC) Detection IHC kit (Abcam, Cambridge, UK) according to the manufacturer’s protocols. As the primary antibody, the human nuclear antigen antibody (1:200, Abcam) was diluted and incubated for 1.5 h at room temperature.

### 2.16. Statistical Analysis 

All quantitative data were presented as the mean ± standard error of the mean from three independent experiments. Statistical data were analyzed using SigmaPlot 12 software. Comparisons between two experimental groups were performed using the two-tailed Student’s *t*-test. Multiple experimental groups were assessed using a one-way analysis of variance. A *p*-value < 0.05 was considered statistically significant.

## 3. Results

### 3.1. Effect of High Glucose on mtROS Accumulation and Apoptosis

**Figure 1:**
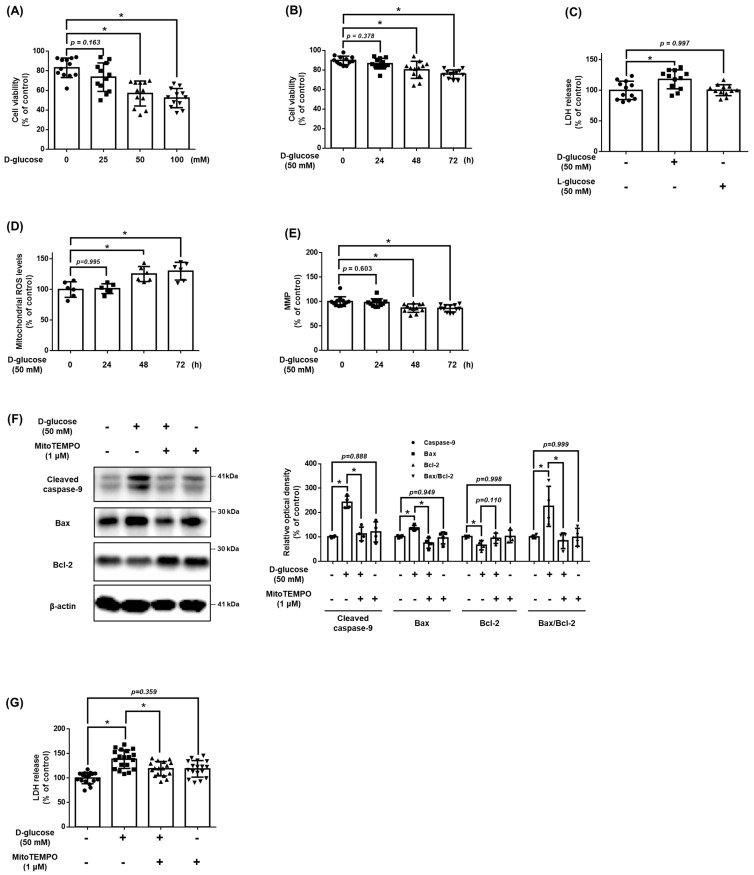
Effect of high glucose on mitochondrial ROS accumulation and apoptosis of MSCs. (**A**) UCB-MSCs were treated with various concentrations (0–100 mM) of D-glucose for 48 h. Cell viability was measured by trypan blue exclusion assay. (**B**) UCB-MSCs were treated with D-glucose (50 mM) at various times (0–72 h). Cell viability was measured by trypan blue exclusion assay. (**C**) UCB-MSCs were treated with 50 mM of D-glucose or 50 mM of L-glucose for 48 h. Cytotoxicity in UCB-MSC-conditioned medium was detected by an LDH release detection kit. (**D**) UCB-MSCs were treated with D-glucose (50 mM) at various times (0–72 h). Mitochondrial ROS level was assessed by MitoSOX staining. (**E**) Mitochondrial membrane potential was assessed by TMRE staining. (**F**) UCB-MSCs were pretreated with MitoTEMPO (1 μM) for 30 min prior to D-glucose (50 mM) for 48 h. Protein expression levels of Bax, Bcl-2, and cleavage caspase-9 were confirmed by western blot analysis. (**G**) UCB-MSCs were pretreated with MitoTEMPO (1 μM) for 30 min prior to D-glucose (50 mM) for 48 h. Cytotoxicity in UCB-MSC-conditioned medium was detected by an LDH release detection kit. * indicates *p* < 0.05.

To identify the effect of D-glucose on UCB-MSC viability, we treated the UCB-MSCs with D-glucose in a dose and time-dependent manner. Cell viability of UCB-MSCs was significantly reduced after 48 h and at concentrations greater than 50 mM of D-glucose (Figure 1A,B). To determine whether the osmotic pressure was the cause of cell death, we evaluated the cytotoxicity levels. The LDH release levels were increased with increased D-glucose but were not affected with increased L-glucose (Figure 1C). The UCB-MSCs treated with D-glucose for more than 48 h had a significantly increased mtROS compared to the UCB-MSCs treated with D-glucose for less than 48 h or the non-treated UCB-MSCs (Figure 1D). In addition, MMP decreased during high glucose treatment (Figure 1E). These results indicate that high glucose-induced cell death through mitochondrial dysfunction. However, treatment with the MitoTEMPO, mtROS inhibitor, reversed the increased cytotoxicity and decreased expression of cleaved caspase-9 and Bax under high glucose conditions (Figure 1F,G). Therefore, we confirmed that high glucose caused cell death, and the main cause of high glucose-induced apoptosis was mtROS.

### 3.2. Effect of High Glucose-Induced OGT on Cellular Ca^2+^ Accumulation and NFATC1 Signaling Activation

**Figure 2:**
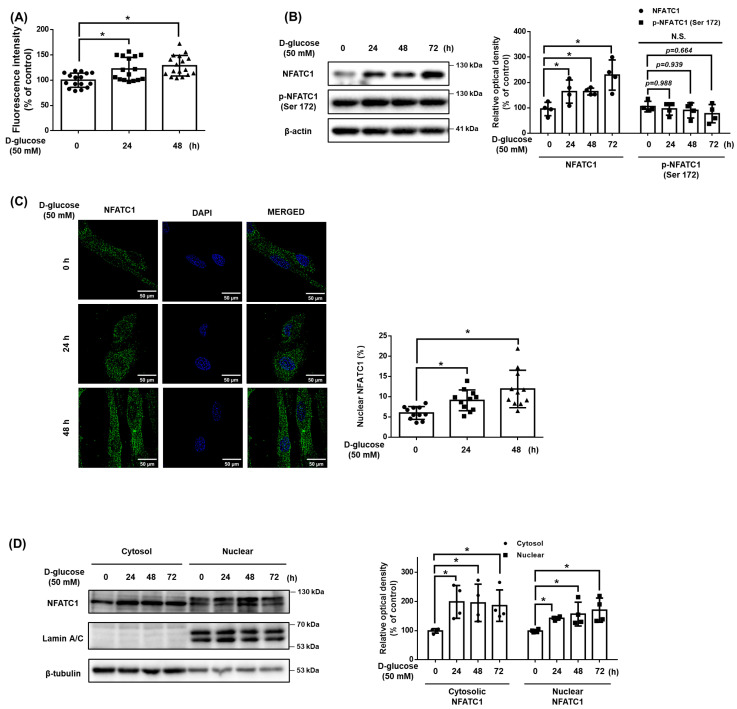
Effect of high glucose induced OGT on cellular Ca^2+^ accumulation and NFATC1 signaling activation. (**A**) UCB-MSCs were incubated with D-glucose (50 mM) for time-lapse (0–48 h). Fluo-3, AM for 30 min prior to Ca^2+^ influx measurement. (**B**) UCB-MSCs were incubated with D-glucose (50 mM) for time-lapse (0–72 h). Protein expression levels of NFATC1 and p-NFATC1 were confirmed by western blot analysis. (**C**) UCB-MSCs were treated with D-glucose (50 mM) at various times (0–48 h). Cells were immunostained with an anti-NFATC1 antibody (green). The nucleus is counterstained with DAPI (blue). Magnification ×1000. (**D**) UCB-MSCs were treated with D-glucose (50 mM) at various times (0–48 h). Subcellular fractions of cytosol and nucleus were analyzed with western blot analysis. N.S. Not significant. * indicates *p* < 0.05.

To confirm whether there was high glucose at the intracellular Ca^2+^ level in UCB-MSCs, the Ca^2+^ level and the expression of NFATC1 activated by Ca^2+^ were confirmed. After a time-dependent treatment with high glucose 50 mM, there was a significant change in Ca^2+^ level from 24 h (Figure 2A). In addition, the protein expression levels of the dephosphorylated NFATC1, active form, were increased at 24 h, but the expression of the phosphorylated NFATC1 at Ser172 (p-NFATC1^ser172^), inactive form, was not increased (Figure 2B). When NFATC1 is activated, it is translocated into the nucleus and functions as a transcription factor. Nuclear fractionation and immunocytochemistry results showed that the expression level of activated NFATC1 was increased in both the cytosol and nucleus and that the expression level of NFATC1 in the nucleus/total cell was enhanced (Figure 2C,D). This result indicates that the dephosphorylated and accumulated NFATC1 was translocated into the nucleus.

### 3.3. Role of O-GlcNAcylation in High Glucose Conditions

**Figure 3:**
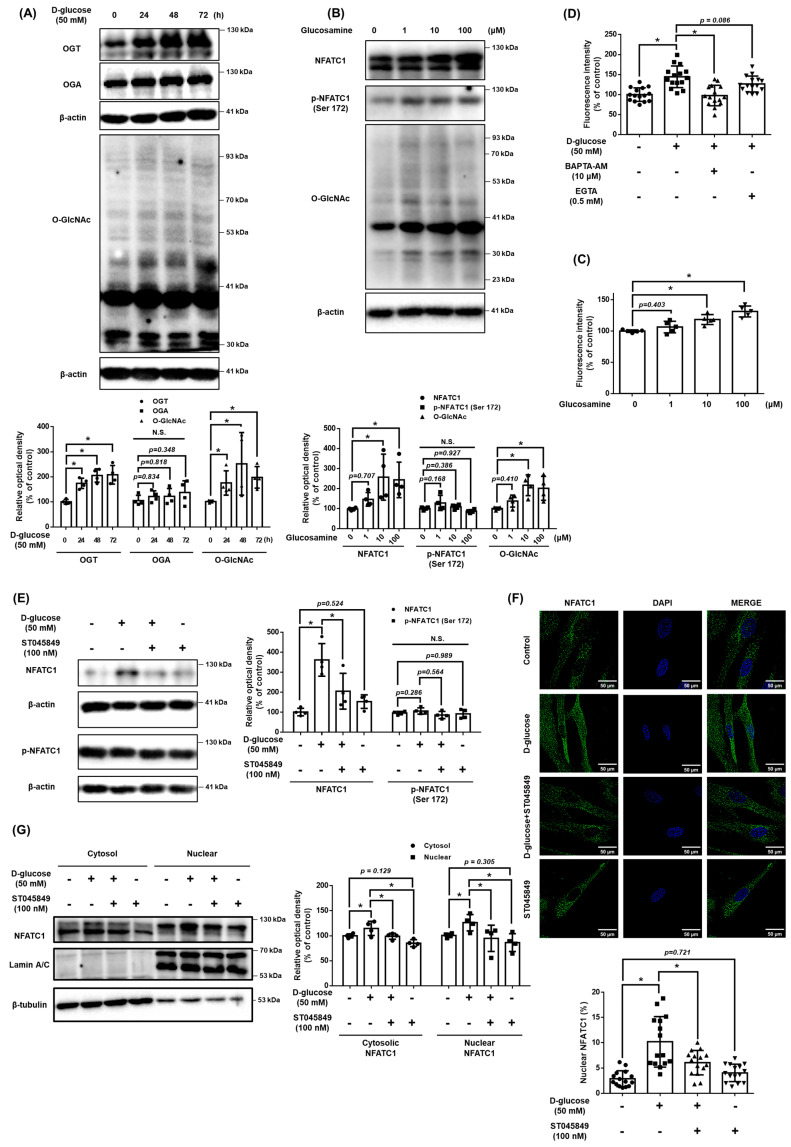
Role of O-GlcNAcylation in high glucose-mediated NFATC1 activation and intracellular Ca^2+^ release. (**A**) UCB-MSCs were incubated with D-glucose (50 mM) for time-lapse (0–72 h). Protein expression levels of OGT, OGA, and O-GlcNAc were confirmed by western blot analysis. (**B**) UCB-MSCs were incubated with glucosamine dose (0–100 μM) for 24 h. The protein expression level of O-GlcNAc was confirmed by western blot analysis. (**C**) UCB-MSCs were incubated with glucosamine dose (0–100 μM) for 24 h. Fluo-3, AM staining. (**D**) UCB-MSCs were incubated with D-glucose (50 μM) for 24 h. Cells were incubated in media pretreated with EGTA (0.5 mM, Ca^2+^ chelator) and BAPTA-AM (10 μM, intracellular Ca^2+^ chelator) before 30 min treating Fluo-3, AM solution. Change of intracellular Ca^2+^ in the cytosol was determined by Fluo-3-AM (3 μM) staining. (**E**) UCB-MSCs were pretreated with ST045849 (100 nM) for 30 min prior to D-glucose (50 mM) for 24 h. Protein expression levels of NFATC1 and p-NFATC1 were confirmed by western blot analysis. (**F**) UCB-MSCs were pretreated with ST045849 (100 nM) for 30 min prior to D-glucose (50 mM) for 24 h. Cells were immunostained with an anti-NFATC1 antibody (green). The nucleus is counterstained with DAPI (blue). Magnification ×1000. (**G**) UCB-MSCs were pretreated with ST045849 (100 nM) for 30 min prior to D-glucose (50 mM) for 24 h. Subcellular fractions of cytosol and nucleus were analyzed with western blot analysis. N.S. Not significant. * indicates *p* < 0.05.

We investigated O-GlcNAcylation as a regulator of intracellular Ca^2+^ levels in high glucose conditions. When treated with high glucose in a time-dependent manner, the expression of OGT and O-GlcNAc were increased, and the expression of OGA was not significantly changed (Figure 3A). In addition, When treated glucosamine at 10 μM or more, the expression levels of NFATC1, O-GlcNAcylation, and Ca^2+^ concentrations increased. However, the expression of p-NFATC1^ser172^ was not significantly changed (Figure 3B,C). These results indicate that an increase in O-GlcNAcylation affects the Ca^2+^ level and activation of NFATC1. To investigate the high glucose-induced Ca^2+^ inflow pathway, we pretreated cells with EGTA, an extracellular Ca^2+^ inhibitor, and BAPTA, an intracellular Ca^2+^ inhibitor, for 30 min prior to high glucose treatment. Our data shows that the intracellular Ca^2+^ levels increased by high glucose conditions were reversed with BAPTA pretreatment but not with EGTA pretreatment. This finding indicates that high glucose conditions induce intracellular Ca^2+^ accumulation (Figure 3D). High glucose conditions increased the activity of NFATC1, but the treatment of ST045849, an OGT inhibitor, inhibited the expression of high glucose-induced NFATC1. In addition, neither high glucose conditions nor ST045849 affected the expression of p-NFATC1^ser172^ (Figure 3E). Immunocytochemistry and nuclear fractionation were conducted to verify whether the increased O-GlcNAcylation by high glucose conditions affected the activity and nucleus translocation of NFATC1. It was confirmed that the nuclear translocation of NFATC1 increased in high glucose conditions and decreased with ST045849 pretreatment (Figure 3F,G). These results indicate that high glucose conditions activate OGT, which leads to intracellular Ca^2+^ levels and the nuclear translocation of NFATC1 in UCB-MSCs.

### 3.4. Effect of Tacrolimus on High Glucose-Induced Mitochondrial Oxidative Stress and Cell Viability

**Figure 4:**
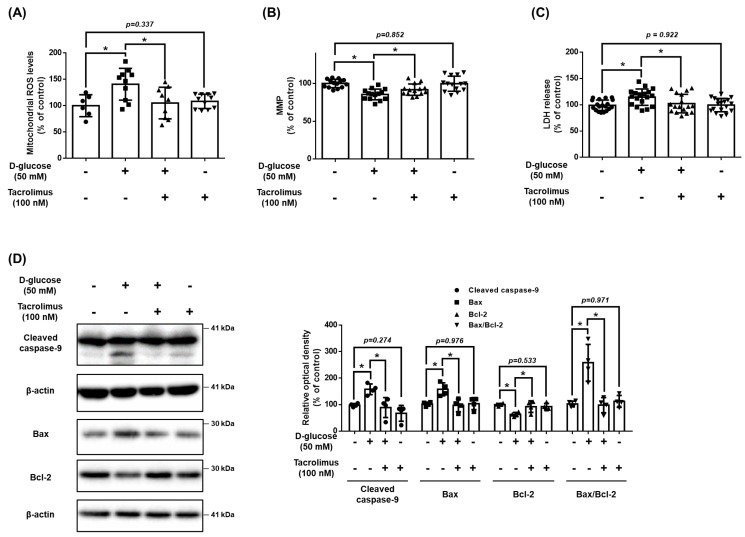
Effect of tacrolimus on high glucose induced-mitochondrial oxidative stress. (**A**–**D**) UCB-MSCs were pretreated with tacrolimus (100 nM) for 30 min prior to D-glucose (50 mM) treatment for 48 h. (**A**) Mitochondrial ROS level was assessed by MitoSOX staining. (**B**) Mitochondrial membrane potential was assessed by TMRE staining. (**C**) Cytotoxicity in UCB-MSC-conditioned medium was detected by an LDH detection kit. (**D**) The protein expression levels of Bax, Bcl-2, and cleaved caspase-9 were confirmed by western blot analysis. * indicates *p* < 0.05.

We investigated the effect of tacrolimus on mitochondrial oxidative stress and cell death induced by high glucose conditions. Tacrolimus (100 nM) reduced the levels of high glucose-induced mtROS (Figure 4A). Results from the TMRE staining assay indicated that high glucose decreased the MMP, and cell viability was recovered by tacrolimus pretreatment (Figure 4B,C). The pro-apoptotic protein Bax and cleaved caspase-9 expressions were increased by high glucose but were recovered by tacrolimus pretreatment (Figure 4D). These results suggest that pretreatment with tacrolimus alleviates the mitochondrial oxidative stress induced by high glucose conditions. 

### 3.5. Protective Effect of Tacrolimus Pretreated UCB-MSCs in DR

**Figure 5:**
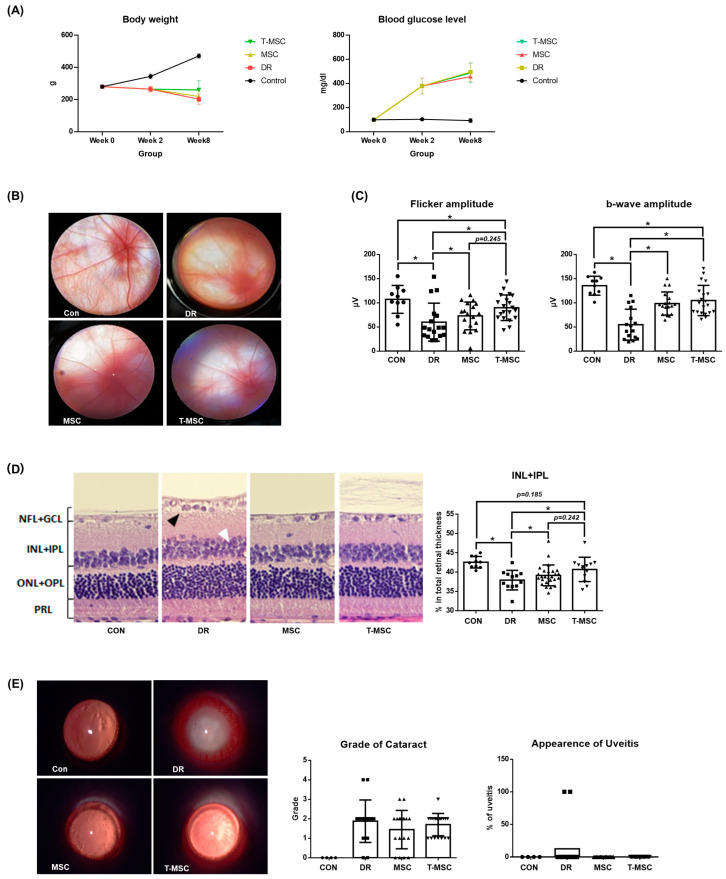
Therapeutic effect of tacrolimus-pretreated MSC transplantation on DR rat model. (**A**) Body weight and blood glucose level. No significant differences were observed between the DR, MSC, and T-MSC groups in body weight and blood glucose level at 8 weeks after STZ injection. (**B**) Fundus images. Attenuated retinal vessels were observed in the DR group. Retinal vessels were observed more clearly in the MSC and T-MSC groups. Magnification ×16. (**C**) Flicker and flicker electroretinography. The retinal function deterioration of early DR was shown by decreased amplitude in the DR group in flicker ERG. Decreased amplitude of b-wave in the DR group shows deterioration of retinal function in early DR. (**D**) H&E stain of the retina ×40. Edematous change, vacuolar degeneration of the ganglion cell layer (black arrow), and non-uniform arrangement of the inner and outer nuclear layers (white arrow) are observed in the DR group. The INL + IPL ratio in the DR group was smaller than in the control group, and it was higher in the MSC and T-MSC groups. (**E**) Slit lamp biomicroscopic examination. Uveitis was observed in 12.5% of the DR group. Magnification ×16. * indicates *p* < 0.05. Abbreviations: CON, control; DR, diabetes retinopathy; MSC, mesenchymal stem cells, T-MSC, tacrolimus-pretreated MSC.

The body weight and blood glucose levels of the rats were measured at 0, 2, and 8 weeks after STZ injection. At 2 weeks after STZ injection, blood glucose levels of 250 mg/dL or higher were observed in the STZ injection groups, and weight loss was also observed. After 8 weeks of injection, a greater difference in body weight and blood glucose levels was observed between the control and diabetic groups. Compared to the DR group, the MSC and T-MSC groups had no significant changes in their body weight and blood glucose levels (Figure 5A). In all rats pretreated with tacrolimus, clinical symptoms related to systemic immunosuppression were not observed. In the DR group, the fundus was blurred due to the formation of cataracts, and the thickness of the retinal blood vessels was reduced. On the other hand, retinal vessels were more clearly observed and thicker in the fundus of the MSC and T-MSC groups compared to the DR group (Figure 5B). To evaluate the function of the cone photoreceptor cells and inner retinal layer, flicker ERG and b-wave amplitude were measured. Compared with the control group, the flicker and b-wave amplitude were significantly reduced in the DR group, but both the MSC and T-MSC groups showed improvement. In addition, the T-MSC group showed greater improvement than the MSC group—in both the flashes and flicker ERGs (Figure 5C). The measure of tissue change showed that the ratio of INL + IPL was decreased in the DR group but was alleviated in the MSC and T-MSC groups (Figure 5D). The effect was greater in the inner retina, and we observed that the protective effect was further enhanced with tacrolimus pretreatment. A slit lamp examination was performed to observe other ocular changes. Cataract formation was not significantly different between the three diabetes groups. With signs of conjunctival hyperemia and iris synechiae, the DR group had uveitis, but no signs of ocular inflammation were observed in the MSC and T-MSC groups. A significant decrease in the incidence of uveitis was confirmed in the injection groups (Figure 5E). These results demonstrate that MSCs have anti-inflammatory properties in the ocular tissue, although they did not prevent the formation of diabetes-induced cataracts.

### 3.6. Role of NFATC1 in DRP1 and Mitochondrial Fission under High Glucose Conditions 

**Figure 6:**
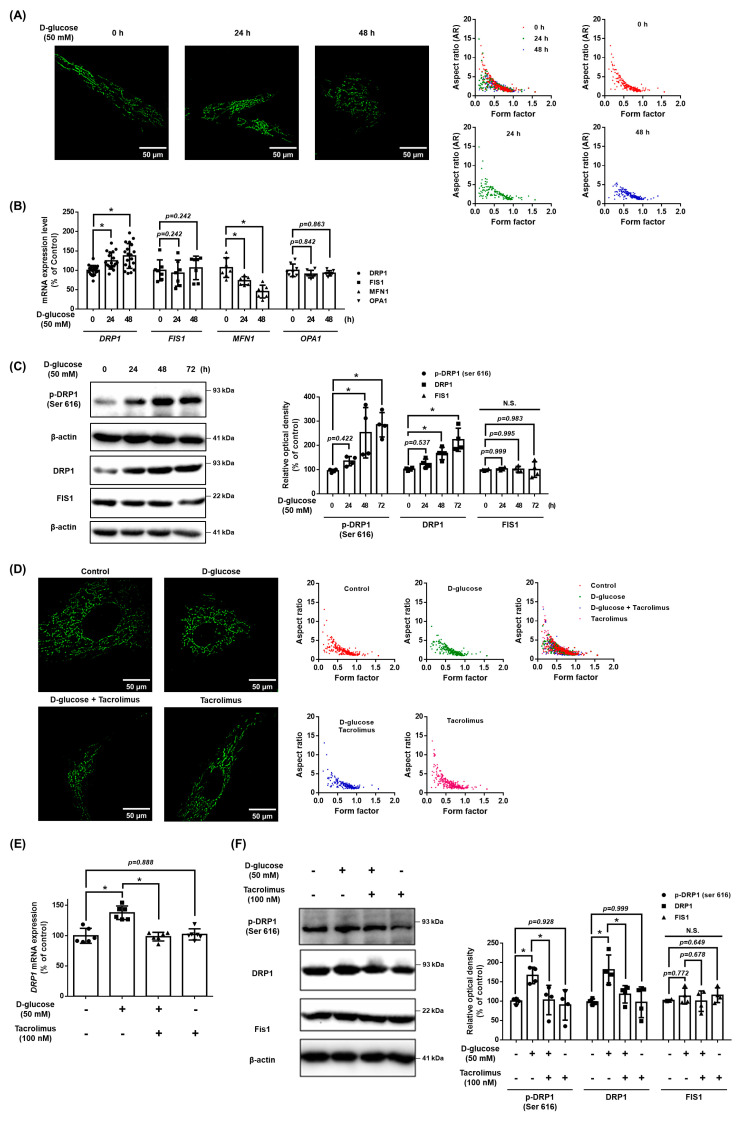
Role of NFATC1 in DRP1 and mitochondrial fission under high glucose. (**A**) UCB-MSCs were treated with D-glucose (50 mM) at various times (0–48 h). Cells were stained with Mitotracker. Magnification ×1000. (**B**) UCB-MSCs were treated with D-glucose (50 mM) at various times (0–48 h). The mRNA expression levels of *DRP1*, *FIS1*, *OPA1*, and *MFN1* were analyzed using quantitative real-time PCR (qPCR). (**C**) UCB-MSCs were treated with D-glucose (50 mM) treatment for time-lapse (0–48 h). Protein expression levels of DRP1, p-DRP1^ser616^, and Fis1 were confirmed by western blot analysis. (**D**) UCB-MSCs were pretreated with tacrolimus (100 nM) for 30 min prior to D-glucose (50 mM) treatment for 48 h. Cells were stained with Mitotracker. Magnification ×1000. (**E**) UCB-MSCs were pretreated with tacrolimus (100 nM) for 30 min prior to D-glucose (50 mM) for 48 h. The mRNA expression levels of *DRP1* and *FIS1* were analyzed using qPCR. (**F**) UCB-MSCs were pretreated with tacrolimus (100 nM) for 30 min prior to D-glucose (50 mM) treatment for 48 h. Protein expression levels of DRP1, p-DRP1^ser616^, and Fis1 were confirmed by western blot analysis. N.S. Not significant. * indicates *p* < 0.05.

We investigated the role of NFATC1 in the mitochondrial dynamics of UCB-MSCs under high glucose conditions. The results demonstrate that high glucose conditions stimulated mitochondrial fission in a time-dependent manner (Figure 6A). In addition, high glucose conditions stimulated the increase in the expression level of *DRP1* mRNA but decreased the *MFN1* mRNA expression levels. Meanwhile, high glucose conditions did not affect the mRNA expression levels of *FIS1* and *OPA1* (Figure 6B). The protein expression of DRP1 increased with high glucose conditions in a time-dependent manner and increased the expression of phosphorylated DRP1 at Ser616 (p-DRP1^ser616^) but not FIS1 (Figure 6C). Furthermore, we investigated the mitochondrial morphology and expression levels of the mitochondrial dynamics factors to identify the regulatory effect of tacrolimus on the mitochondrial dynamics under high glucose conditions. The high glucose induced a decrease in the aspect ratio and morphology of the MSCs, but these mitochondrial changes were inhibited with tacrolimus pretreatment (Figure 6D). We found that the DNML1L_1 promoter contains an NFATC1 binding site located between the transcription start site and a point 500 bp upstream (Supplementary file: Figure S1). High glucose increased the *DRP1* mRNA expression levels, which were reversed with tacrolimus pretreatment (Figure 6E). The protein expression of DRP1 was increased in high glucose conditions, and the activity of p-DRP1^ser616^ was substantially increased. Tacrolimus pretreatment decreased the phosphorylation of DRP1, which was induced by high glucose conditions (Figure 6F). Therefore, we found that tacrolimus pretreatment prevented the high glucose-induced mitochondrial fission of UCB-MSCs.

### 3.7. Role of NFATC1-Induced DRP1 Activation in Mitochondrial Oxidative Stress

**Figure 7:**
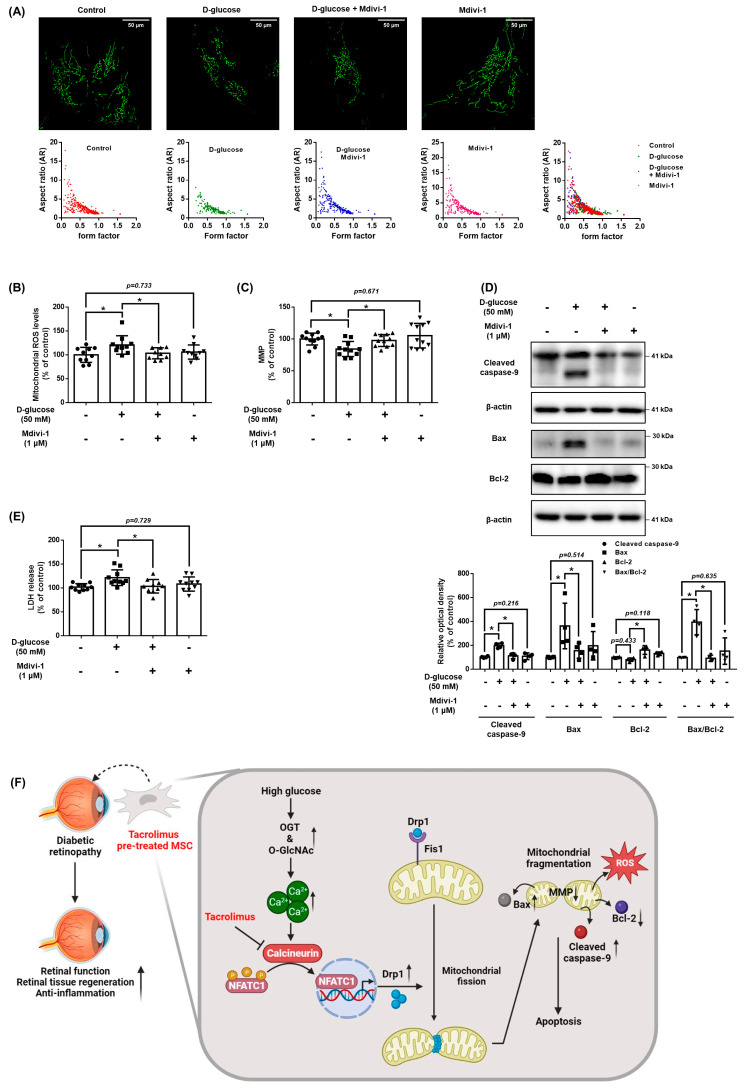
Role of NFATC1-induced DRP1 activation in mitochondrial oxidative stress. (**A**–**E**) UCB-MSCs were pretreated with Mdivi-1 (1 μM) for 30 min prior to D-glucose (50 mM) treatment for 48 h. (**A**) Cells were stained with Mitotracker. Magnification ×1000. (**B**) Mitochondrial membrane potential was assessed by TMRE staining. (**C**) Mitochondrial ROS level was assessed by MitoSOX staining. (**D**) Cytotoxicity in UCB-MSC-conditioned medium was detected by an LDH detection kit. (**E**) The protein expression levels of Bax, Bcl-2, and cleaved caspase-9 were confirmed by western blot analysis. * indicates *p* < 0.05. (**F**) The schematic model for the action mechanism of tacrolimus on UCB-MSCs under high glucose conditions. High glucose stimulates intracellular Ca^2+^ release through an increase in O-GlcNAcylation, which leads to activation of NFATC1. Activated NFATC1 increases the mRNA and protein expression of DRP1, leading to mitochondrial fission. Excessive mitochondrial fission generates mtROS, which causes cell death. Tacrolimus inhibits the activity of NFATC1 induced by excess Ca^2+^, thereby reducing high glucose-induced mitochondrial fission and mtROS, leading to resistance to cell death. In conclusion, tacrolimus enhances the therapeutic potential of UCB-MSCs through the inhibition of mitochondrial fission by suppressing high glucose-induced NFACT1 activity.

We investigated the role of DRP1 in mitochondrial oxidative stress and apoptosis of UCB-MSCs under high glucose conditions. Mitochondrial morphology showed that high glucose induced a decrease in the aspect ratio and morphology of the MSCs, but these mitochondrial changes were inhibited by pretreatment with Mdivi-1 (Figure 7A). TMRE staining results showed that Mdivi-1, a DRP1 inhibitor, increased the MMP, which was reduced by high glucose (Figure 7B). In addition, Mdivi-1 pretreatment reduced the high glucose and increased mtROS levels and LDH release levels (Figure 7C,D). From the western blot analysis data, it can be seen that Mdivi-1 pretreatment prevented the expression of the high glucose-increased pro-apoptotic proteins, such as cleaved caspase-9 and Bax (Figure 7E). Therefore, these results indicate that DRP1-mediated mitochondrial fission is closely associated with mitochondrial oxidative stress and UCB-MSCs apoptosis under high glucose conditions. 

## 4. Discussion

The present study highlighted that high glucose conditions induced intracellular Ca^2+^ levels through O-GlcNAcylation and the regulatory effect of tacrolimus on the nuclear translocation of NFATC1 and DRP1 expression in UCB-MSCs under high glucose conditions. In addition, hyperglycemia induced the accumulation of mtROS, resulting in mitochondrial dysfunction (Figure 7F). This defect appears as a decrease in the MMP and a change in the mitochondrial morphology and dysfunction, which is closely associated with high glucose-stimulated apoptosis. Previous studies have reported that the repression of the antioxidant defense system by hyperglycemia-induced epigenetic modification also leads to the imbalance between the removal and generation of ROS [30]. Excessive accumulation of ROS induces mitochondrial damage, cellular apoptosis, inflammation, lipid peroxidation, and structural and functional alterations in the retina [30]. Similar to our research, another study reported that cell viability was significantly reduced in MSCs cultured with serum from patients with type 2 diabetes [31]. This indicates that under oxidative stress, MSCs are highly susceptible to apoptosis, and their function is decreased. Moreover, cell condition control, such as the removal of the harsh microenvironment during culture and transplantation, appropriate to the situation maintains the function of the MSCs and improves their efficacy as therapeutic agents [32]. Consequently, we demonstrated that mitochondrial stress regulation is important for regeneration and transplanted cell survival under high glucose conditions.

According to several previously reported studies, the excessive uptake of Ca^2+^ in the mitochondria was converted during a potentially harmful mechanism, leading to apoptosis. For example, excessive Ca^2+^ activates neuronal nitric oxide synthase and generates NO through calcineurin-induced phosphorylation, inducing mitochondrial permeability transition pore (mPTP) opening and glutamate-induced mitochondrial depolarization [11]. In addition, high glucose conditions increase the expression of junction proteins between the mitochondria and endoplasmic reticulum (ER), increasing the Ca^2+^ inflow from the ER to the mitochondria, leading to the generation of mtROS [33]. A previous study also found that high glucose conditions increased the Ca^2+^ in cells through the Orai1 Ca^2+^ channel [34]. Consistent with previous findings, our data demonstrated that high glucose conditions induce excessive Ca^2+^ accumulation and cause mitochondrial disorders. Therefore, previous and present findings clarify the role of Ca^2+^ signaling as a regulator in mitochondrial stress and dysfunction in UCB-MSCs under high glucose conditions. Excessive Ca^2+^ level is reported in oxygen-glucose deprivation conditions rather than in high glucose conditions, resulting in the activation of calcineurin [35]. However, our finding revealed that high glucose conditions stimulated excessive Ca^2+^ levels, and high glucose-induced intracellular Ca^2+^ level was caused by O-GlcNAcylation. In support of our findings, several studies have reported that continuous hyperglycemic conditions in diabetes increased glutamine fructose-6-phosphate amidotransferase and increased O-GlcNAc-modified proteins via hexosamine biosynthesis pathway (HBP), which leads to cellular oxidative stress [28,36,37]. Therefore, HBP was chronically active in diabetes, and protein O-GlcNAcylation was associated with complications of diabetes [38]. However, the role of O-GlcNAcylation in intracellular Ca^2+^ regulation appears controversial. The present study demonstrated that high glucose conditions increase the inflow of intracellular Ca^2+^ through OGT activation, which is consistent with a previous finding that O-GlcNAcylation plays a key role in Ca^2+^/calmodulin-dependent kinase IV activation [39]. Meanwhile, other studies have reported that O-GlcNAcylation-modified STIM-1 inhibited the store-operated Ca^2+^ ion entry via the dephosphorylation of STIM-1 at Ser 621 in HEK cells [40]. Therefore, further investigations are required to confirm the role of O-GlcNAcylation in intracellular Ca^2+^ regulating protein activity in various cell types.

The present study demonstrated that high glucose-induced excessive Ca^2+^ level increases the NFATC1 activity. Moreover, NFATC1 activation is regulated by a variety of factors, including cyclosporin A (CsA) and FK506, which inhibit calcineurin by binding to the calcineurin subunit B (CnB) domain of calcineurin. In addition, Down’s syndrome candidate region 1 (DSCR1), calcineurin-binging protein 1 (CABIN1), and A-kinase anchor protein 79 (AKAP79) regulate the activity of NFAT by inhibiting calcineurin [41]. A previous study demonstrated that DSCR1 (Adapt78 or RCAN1) regulates glucagon synthesis in pancreatic islet cells and modulates blood glucose levels [42]. In addition, the knockout of AKAP 150 (murine AKAP150, a homolog of human AKAP79) was reported to ameliorate vascular dysfunction in diabetes by improving BK channels through the Akt/GSK3β signaling pathway [43].

Consistent with our findings, a continuous increase in intracellular Ca^2+^ levels activates calcineurin/NFAT signaling, which leads to apoptosis induced by fission and dysfunction of mitochondria [44]. Moreover, MSC transplantation with tacrolimus co-treatment in patients with kidney transplantation significantly alleviated the host versus graft rejection response and increased the organ life span [45]. Concerning previous studies that demonstrated the role of NFATC1 in MSC differentiation into osteoclasts and chondrocytes, tacrolimus-inhibited NFATC1 signaling is an effective strategy to maintain the paracrine effect of undifferentiated MSC for tissue regeneration [46,47,48]. Although the regulatory effect of tacrolimus pretreatment on the paracrine effect of MSCs in vitro requires further study, the present study suggested that the increased survival rate of tacrolimus pretreated MSCs under high glucose is associated with upregulation of its paracrine and tissue regenerative capacity of transplanted MSC. In previous studies, the improvement in retinal function of a single MSC administration evaluated through ERG lasted for 1–4 weeks [18,21]. Based on these studies, when MSCs were injected every 2 weeks, the amplitude of the ERG indicating retinal function was increased in both the MSC and T-MSC groups compared to the DR group. However, body weight and blood glucose levels were not significantly different in the MSC and T-MSC groups compared to the DR group. This demonstrates that the subconjunctival MSCs administration has protective effects on the early DR process but has no systemic effect.

In this experiment, we investigated the preventive effect of DR by MSC subconjunctival injection, which is less invasive than intravitreal or subretinal injections, with multiple administrations. Intravitreal and subretinal injections have generally been used for research purposes to treat retinal diseases. Although it has the advantage of acting directly on the retina, it can lead to complications such as cataract development, endophthalmitis, retinal or vitreous hemorrhage, retinal tears and detachments, pain, and acute intraocular pressure elevation [49,50,51]. A previous rat study reported that intravitreal injection of MSCs caused retinal blood vessel degeneration, cataracts, and inflammation [22]. On the other hand, subconjunctival injection is safe, easier, and less painful than intravitreal injection and can only be performed under topical anesthesia. In addition, complications such as retinal detachment, intraocular hemorrhage, and cataracts occur less frequently. In addition, subconjunctival injection also has a medicinal effect on the posterior segment [52,53]. In immunohistochemistry, the MSCs were rarely observed in the retina in both the MSC and T-MSC groups. Therefore, the migration of the MSCs to the retinal tissue was not a major mechanism of prevention of DR. The protective effects of the subconjunctival MSCs in this study might be paracrine effects. In future studies, the secreted substances that have protective effects on DR should be investigated. In the T-MSC group, the result of ERG, especially the b-wave amplitude, indicated that the whole retinal function was better than that of the MSC group. This indicates the retinal protective effects of the tacrolimus-pretreated MSCs on DR-impaired retinal function. Additional research is required to determine whether tacrolimus pretreatment can be applied to various disease treatments. Periodic subconjunctival MSC injection showed protective effects against DR in the retina of diabetic rats. In addition, the pretreatment with tacrolimus can increase the therapeutic effect of MSCs, thus more efficiently preventing DR. 

However, although there are several reports in which MSCs and tacrolimus have been studied for immunotherapy purposes, there have been no reports of using them to develop metabolic treatments. In our research, regulation of mitochondrial fission by Mdivi-1 significantly prevented the high glucose-induced mtROS, MMP down-regulation, and apoptosis in UCB-MSCs. In addition, a common feature of mitochondria in hyperglycemia is fragmentation due to the activity of DRP1 and the downregulation of MFN2 [54]. The morphology of mitochondria is also controlled by Ca^2+^, which causes fragmentation of mitochondria [55]. Cells with a fragmented network have a lower MMP due to limited electron transport and proton pump action and a higher rate of mitophagy due to an increased number of dysfunctional mitochondria [56]. It has been reported that the anti-diabetic drug dipeptidyl peptidase 4 inhibitor vildagliptin reduced the expression of DRP1 and Fis1, blocked the movement of DRP1 to the mitochondria, and slowed mitochondrial fragmentation induced by hyperglycemia [57]. According to previous studies, calcineurin directly interacts with DRP1 for the dephosphorylation of the Ser 637 residue but not the Ser 616 residue, which is critical for DRP1 activation [58,59,60]. Similarly, we found that p-DRP1 increased with the total DRP1 accumulation. Moreover, we showed that the tacrolimus suppressed NFATC1 signaling directly increased the DRP1 transcription level in MSCs under high glucose conditions.

## 5. Conclusions 

In conclusion, we demonstrated that high glucose-stimulated O-GlcNAcylation is responsible for the increase in intracellular Ca^2+^ levels in UCB-MSCs and elucidated the mechanism by which intracellular Ca^2+^-activated NFATC1 increases DRP1-mediated mitochondrial fission and mitochondrial oxidative stress. In addition, we first propose an NFATC1 inhibition by tacrolimus to prevent high glucose-stimulated mitochondrial apoptosis through the suppression of mitochondrial fission. Finally, we suggest the subconjunctival injection of tacrolimus-pretreated UCB-MSCs as a new strategy to improve the therapeutic efficacy of MSC transplantation for DR treatment without tacrolimus-induced systemic immunosuppression.

## Data Availability

Data are included within the article.

## Supplementary Materials

The following supporting information can be downloaded at: https://www.mdpi.com/article/10.3390/antiox12091727/s1, Figure S1: The NFAT binding site in the DNML1L_1 promoter; Figure S2: Full-length gel images for key data in Figure 1F and Figure 2B,D; Figure S3: Full-length gel images for key data in Figure 3A,B,E,G; Figure S4: Full-length gel images for key data in Figure 4D, Figure 6C,F and Figure 7D; Table S1: Sequences of primers used for real-time PCR.

## Abbreviations

α-MEM	MEM alpha
Bax	Bcl-2-associated protein x
Bcl-2	B-cell lymphoma 2
BSA	Bovine serum albumin
Ca^2+^	Calcium
CCCP	Carbonyl cyanide 3-chlorophenylhydrazone
DAPI	4’,6-Diamidino-2-Phenylindole, dihydrochloride
DR	Diabetic retinopathy
DRP1	Dynamin-related protein 1
ER	Endoplasmic reticulum
ERG	Electroretinography
FBS	Fetal bovine serum
FIS1	Mitochondrial fission 1
GCL	Ganglion cell layer
HBP	Hexosamine biosynthesis pathway
INL	Inner nuclear layer
IPL	Inner plexiform layer
LDH	Lactate dehydrogenase
MFN1	Mitofusin-1
MMP	Mitochondrial membrane potential
mPTP	Mitochondrial permeability transition pore
mtROS	Mitochondrial reactive oxygen species
NFAT	Nuclear factor of activated T cells
NFL	Nerve fiber layer
OGA	*O*-*GlcNAcase*
OGT	O-linked N-acetylglucosaminyltransferase
ONL	Outer nuclear layer
OPA	Optic atrophy 1
OPL	Outer plexiform layer
PBS	Phosphate-buffered saline
PBST	PBS containing 0.1% Tween-20
PRL	Photoreceptor layer
PVDF	Polyvinylidene fluoride
STZ	Streptozotocin
TBST	Tris-buffered saline containing 0.1% Tween-20
TMRE	Tetramethylrhodamine, ethyl ester
T-MSC	Tacrolimus-pretreated MSC injection group
UCB-MSCs	Umbilical cord blood-derived mesenchymal stem cells
VEGF	Vascular endothelial growth factor
