# Supplementary material for: Tacrolimus Improves Therapeutic Efficacy of Umbilical Cord Blood-Derived Mesenchymal Stem Cells in Diabetic Retinopathy by Suppressing DRP1-Mediated Mitochondrial Fission
Source: Antioxidants (Basel). 2023 Sep 6;12(9):1727. doi: 10.3390/antiox12091727 (PMC10525315; doi:10.3390/antiox12091727)
Supplement: Supplementary file 1 [file antioxidants-12-01727-s001.zip › antioxidants-2555650-supplementary.pdf]

Title: Tacrolimus improves therapeutic efficacy of umbilical cord blood-derived mesenchymal stem cells in diabetic retinopathy by suppressing DRP1-mediated mitochondrial fission

Hang Hyo Jo[1,2,a], Yeong Seok Goh[3,a], Hye Jih Kim[1,2], Dae Hyun Kim[1,2], Hyemin Kim[3], Jiyi Hwang[3], Ji Seung Jung[3], Nanyoung Kang[3], Sang Eun Park[3], Kyung Mee Park[3,*], and Hyun Jik Lee[1,2,*]

Affiliation

[1]Laboratory of Veterinary Physiology, College of Veterinary Medicine and Veterinary Medicine Center, Chungbuk National University, Cheongju 28644, Korea

[2]Institute for Stem Cell & Regenerative Medicine (ISCRM), Chungbuk National University, Cheongju 28644, Korea

[3]Laboratory of Veterinary Surgery and Ophthalmology, College of Veterinary Medicine, Chungbuk National University, Cheongju 28644, Korea

[a] These authors contributed equally to this work

[*]Corresponing authors are Kyung-Mee Park and Hyun Jik Lee.

***Kyung-Mee Park, D.V.M., Ph.D.**
Laboratory of Veterinary Surgery and Ophthalmology, College of Veterinary Medicine, Chungbuk National University, Cheongju 28644, Korea

Tel: +82-43-261-2985 / E-mail: parkkm@cbnu.ac.kr

***Hyun Jik Lee, D.V.M., Ph.D.**
Laboratory of Veterinary Physiology, College of Veterinary Medicine and Veterinary Medicine Center, Chungbuk National University, Cheongju 28644, Korea

Institute for Stem Cell & Regenerative Medicine (ISCRM), Chungbuk National University, Cheongju 28644, Korea

Tel: +82-43-261-2597 / E-mail: leehyunjik@chungbuk.ac.kr

Running Title: Therapeutic effect of tacrolimus-pretreated MSCs on diabetic retinopathy

Key words: Diabetic retinopathy, Mesenchymal stem cells, Tacrolimus, Mitochondrial dynamics, O-GlcNAc transferase, Apoptosis

-700 GTAATCCCAACACTTTGGGAGGCCGAGGCGGGTGGATCACTAGGTCCGGAGATCGAGACC

-640 ATCCTGGAAAACACAGTGAAACCCCGTCTCTACTAAAAATACAAAAAATTAGCCGGGCGT

-580 AGTGGTGGGTGCCTGTAGTCCCAGCTACTCCGAAGGCTGAGGCAGGAGAATGGCGTGAAC

-520 CCGGGAGGCGGAATTTGCAGTGGGCCGAGATCGCGCCACTGCACTCCAGCCTGGGCGACA

-460 GAGTGAGACTCCGTCTCAAAAAAAAAAAAAAAATAAATAAATAAAAAATAAATAAAAGGA

-400 AGTGACACTGGATAATTAAGAGACACACCATTGCGCCTTTAACACATATTCACACCCAGC

-340 ACTGTCACATCCATCGTGCCTTGAGGACAGGCATAAATTTACGGCCTTCCTCCTGCGCTC

-280 CCGGCCTGCTCCTAGCCTTTGACTAGAGCCTGCGCAGCATCCCAGTCCCAGGCTAGGGCC

-220 CACCGGTGCCGCGCAGCCTCCTGGAAGCTGTGGGGAGTAGCGTTCCTCCGCTCCAGAACT

-160 ACAACTCCCAGCAGGCCTTGCTCCTCTCCACCTCCCTCGCAGCGCATGGCCTGCCGGGAG

-100 GGGGCAGGTAGCCGGCGGGCCCGGTCCAATGGGTGCCGGCTTCCGAGGAGAGGGCGGAGG

-040 AGAGGAGGAAGGAGGCGAACTGTGGGCCCCGGCCCCATTC

Fig. S1. The NFAT binding site in the DNML1L_1 promoter. The NFAT binding site in the DNML1L_1 promoter is shown. The yellow shaded box represents the consensus sequences of NFATC1.

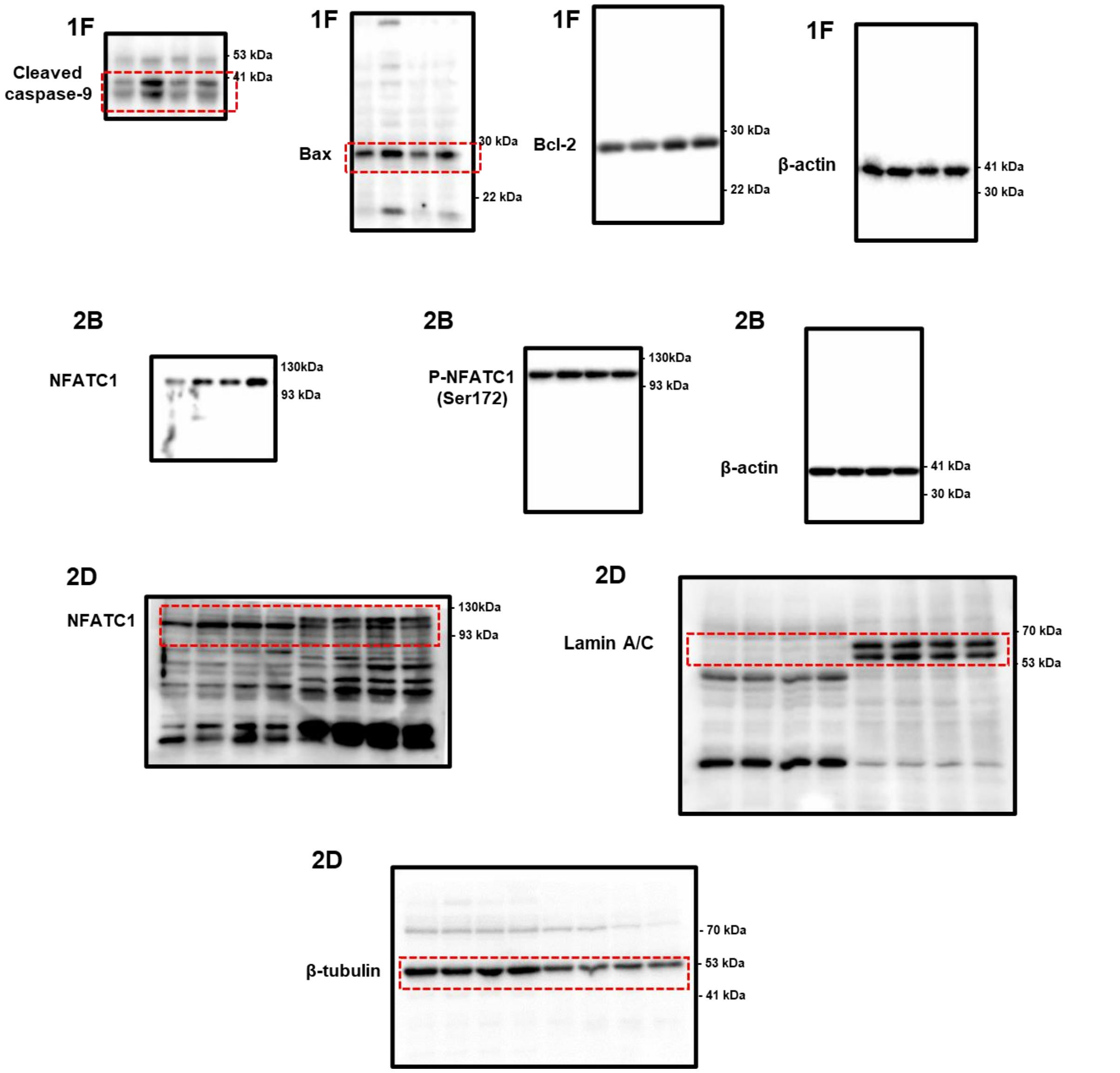

Fig. S2. Full-length gel images for key data in figures 1F-2D. All western blot images are full-length blot images of key blot data in the figures 1F-2D. Dash line box indicates cropped blot image in the figures 1F-2D.

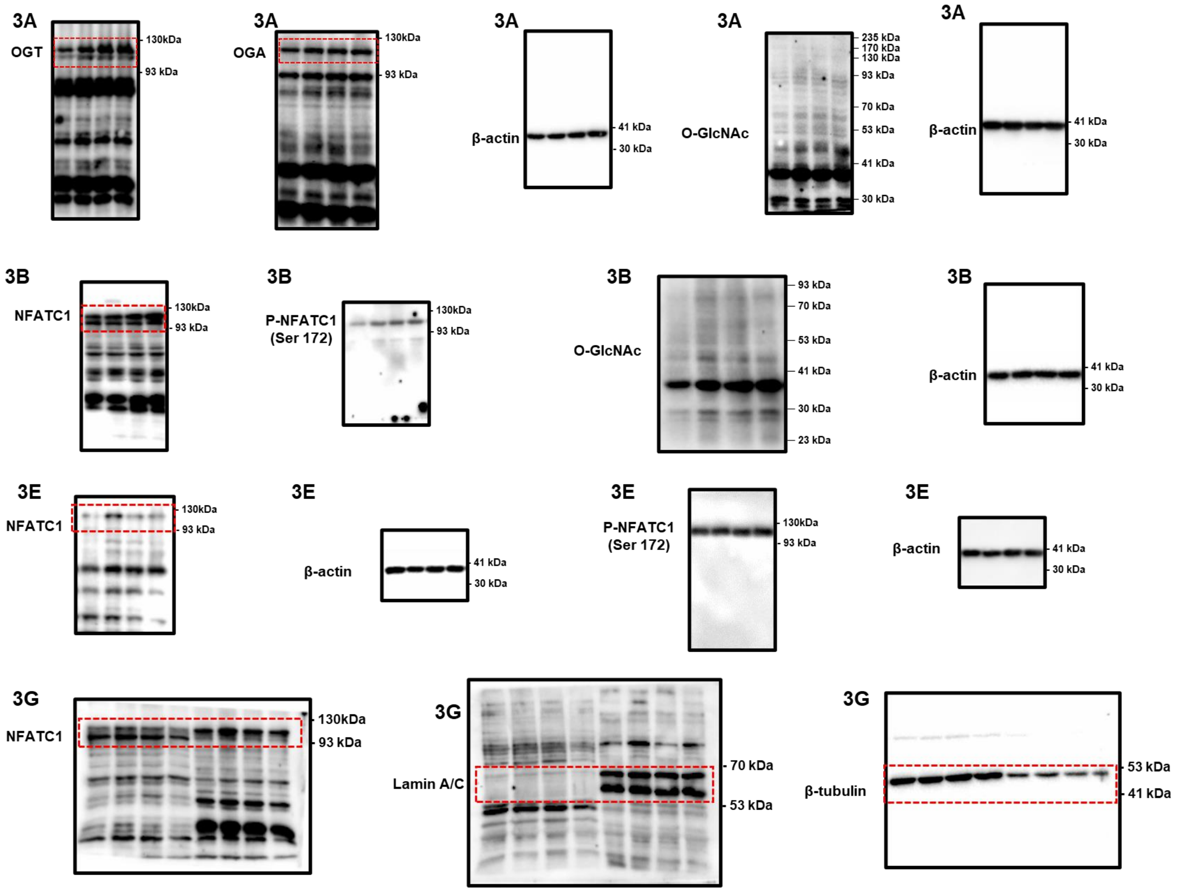

Fig. S3. Full-length gel images for key data in figures 3A-3G. All western blot images are full-length blot images of key blot data in the figures 3A-3G. Dash line box indicates cropped blot image in the figures 3A-3G.

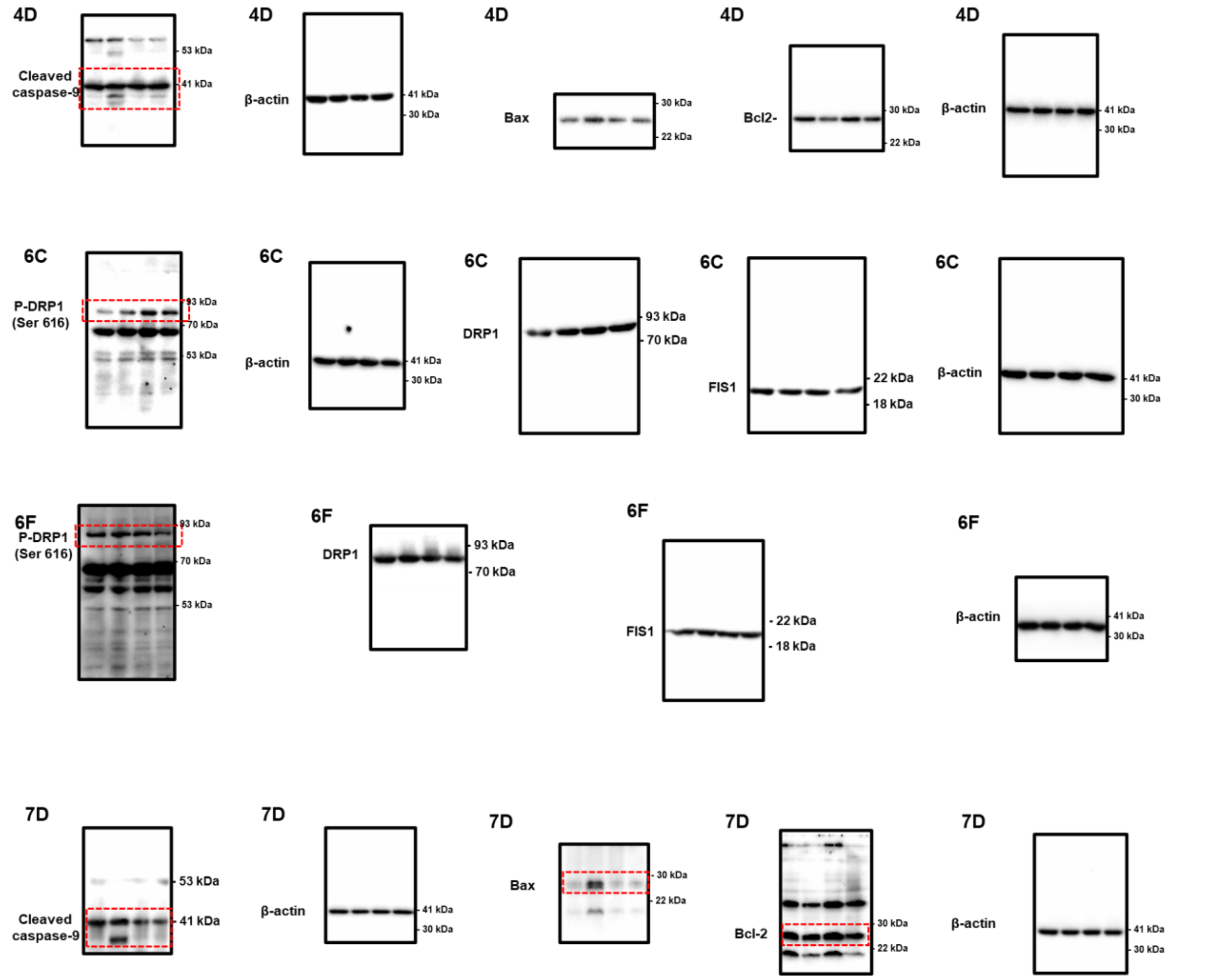

Fig. S4. Full-length gel images for key data in figures 4D-7D. All western blot images are full-length blot images of key blot data in the figures 4D-7D. Dash line box indicates cropped blot image in the figures 4D-7D.

Table. S1. Sequences of primers used for real-time PCR

Gene	Identification	Sequence (5'-3')	Size (bp)	Accession number
DRP1	Sense	CAGTGTGCCAAAGGCAGTAA	212	XM_054370716.1
	Antisense	GATGAGTCTCCCGGATTTCA		
FIS1	Sense	CTTGCTGTGTCCAAGTCCAA	213	XM_054356644.1
	Antisense	CCACAGCCCCGTTTTATTTA		
MFN1	Sense	TTGGAGCGGAGACTTAGCAT	163	NM_033540.3
	Antisense	TTCGATCAAGTTCCGGATTC		
OPA1	Sense	GGCCAGCAAGATTAGCTACG	154	NM_015560.3
	Antisense	ACAATGTCAGGCACAATCCA		
ACTB	Sense	CCACCATGTACCCTGGCATT	189	NM_001101.5
	Antisense	CGGACTCGTCATACTCCTGC		
